# 1-Amino­pyridinium triiodidoplumbate(II)

**DOI:** 10.1107/S1600536810036263

**Published:** 2010-09-15

**Authors:** Shan-Shan Yu, Hua Xian, Hai-Bao Duan

**Affiliations:** a Department of Chemistry, Nanjing Xiaozhuang College, Nanjing 210017, People’s Republic of China

## Abstract

The title complex, (C_5_H_7_N_2_)[PbI_3_], consists of a 1-amino­pyridinium cation, disordered about a mirror plane, and a [PbI_3_]^−^ anion. The Pb^2+^ ion (site symmetry 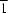) is surrounded by six I atoms in a slightly distorted octa­hedral coordination. The PbI_6_ octa­hedra share faces, building up _∞_^1^[PbI_6/2_] chains running along [010]. The cations are situated between the chains. Coulombic attractions and van der Waals inter­actions between the inorganic and organic components are mainly responsible for the cohesion of the structure.

## Related literature

For background to hybrid materials, see: Rogow *et al.* (2010 ▶); Thirumurugan & Rao (2008 ▶). For structures with lead halide building blocks, see: Li *et al.* (2008 ▶); Zhang *et al.* (2008 ▶).
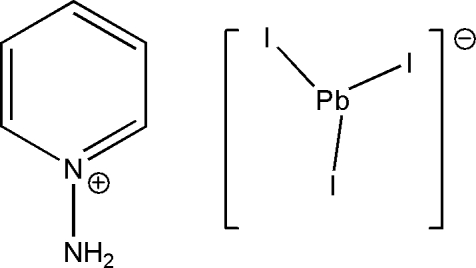


## Experimental

### 

#### Crystal data

(C_5_H_7_N_2_)[PbI_3_]
*M* *_r_* = 683.03
Orthorhombic, 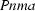

*a* = 15.0417 (19) Å
*b* = 8.1316 (10) Å
*c* = 10.5625 (14) Å
*V* = 1291.9 (3) Å^3^
*Z* = 4
Mo *K*α radiation
μ = 20.18 mm^−1^
*T* = 296 K
0.6 × 0.2 × 0.1 mm

#### Data collection

Bruker SMART CCD area-detector diffractometer
Absorption correction: multi-scan (*SADABS*; Bruker, 2000 ▶) *T*_min_ = 0.011, *T*_max_ = 0.133
10792 measured reflections
1607 independent reflections
1263 reflections with *I* > 2σ(*I*)
*R*_int_ = 0.036

#### Refinement

*R*[*F* ^2^ > 2σ(*F* ^2^)] = 0.025
*wR*(*F* ^2^) = 0.056
*S* = 1.11
1607 reflections
61 parameters
1 restraint
H-atom parameters constrained
Δρ_max_ = 0.86 e Å^−3^
Δρ_min_ = −1.05 e Å^−3^

### 

Data collection: *SMART* (Bruker, 2000 ▶); cell refinement: *SAINT* (Bruker, 2000 ▶); data reduction: *SAINT*; program(s) used to solve structure: *SHELXTL* (Sheldrick, 2008 ▶); program(s) used to refine structure: *SHELXTL*; molecular graphics: *SHELXTL*; software used to prepare material for publication: *SHELXTL*.

## Supplementary Material

Crystal structure: contains datablocks global, I. DOI: 10.1107/S1600536810036263/wm2395sup1.cif

Structure factors: contains datablocks I. DOI: 10.1107/S1600536810036263/wm2395Isup2.hkl

Additional supplementary materials:  crystallographic information; 3D view; checkCIF report

## Figures and Tables

**Table 1:** Selected bond lengths (Å)

Pb1—I3	3.2301 (5)
Pb1—I1	3.2303 (5)
Pb1—I2	3.2412 (5)

**Fig. 1.**
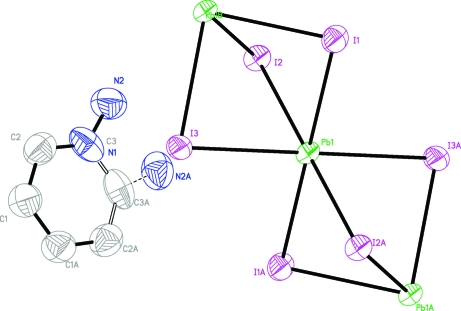
Part of the structure of (I), showing the atom-numbering scheme and displacement ellipsoids at the 30% probability level. Atom C3 and N1 are positionally disordered. H atoms have been omitted for clyrity. [Symmetry code: A x, 0.5 - y, z.]

**Fig. 2.**
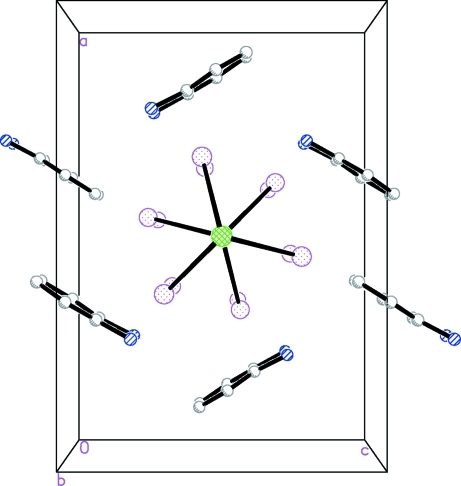
The alignment of inorganic and organic components in the crystals of l along [010].

## supplementary crystallographic information

### Comment

Inorganic metal-halide building-blocks have received special attention with respect to the construction of inorganic-organic hybrid materials (Rogow *et al.*, 2010; Thirumurugan *et al.*, 2008). Among these materials octahedral building blocks of lead halides are frequently found and numerous crystal structures, from one-dimensional chains to three-dimensional frameworks (Li *et al.*, 2008; Zhang *et al.*, 2008), were observed. Herein we report the crystal structure of the title compound, (C_5_H_7_N_2_)[PbI_3_] (I).

Compound (I) crystallizes with one [PbI_3_]^-^ anion and one 1-aminopyridinium cation in the asymmetric unit (Fig. 1). The cation is disordered about a mirror plane. The Pb^2+^ cation is coordinated by six iodide anions in a slightly distorted octahedral coordination geometry. The PbI_6_ octahedra share faces, resulting in anionic chains running along [010]. As show in Fig. 2., the straight inorganic chains are embedded in cationic stacks. Besides Coulomb attractions, only weak van der Waals interactions between the inorganic and organic components exist.

### Experimental

A mixture of PbI_2_ (922 mg, 2.0 mmol) and 1-aminopyridinium iodide (190 mg, 2.0 mmol) in a molar ratio of 1:1 in DMF was slowly evaporated to produce orange needle-shaped crystals.

### Refinement

The H atoms were placed in geometrically idealized positions and refined as riding atoms, with *U*_iso_(H) = 1.2*U*_eq_(C). The cation is disordered about a mirror plane. Atoms C3 and N1 occupy the same site with an occupation factor of 50%. The respective —NH_2_ group and the H atom show likewise half-occupation.

### Crystal data

**Table:**

(C_5_H_7_N_2_)[PbI_3_]	*Z* = 4
*M**_r_* = 683.03	*F*(000) = 1168
Orthorhombic, *P**n**m**a*	*D*_x_ = 3.512 Mg m^−^^3^
Hall symbol: -P 2ac 2n	Mo *K*α radiation, λ = 0.71073 Å
*a* = 15.0417 (19) Å	µ = 20.18 mm^−^^1^
*b* = 8.1316 (10) Å	*T* = 296 K
*c* = 10.5625 (14) Å	Needle, orange
*V* = 1291.9 (3) Å^3^	0.6 × 0.2 × 0.1 mm

### Data collection

**Table:**

Bruker SMART CCD area-detector diffractometer	1607 independent reflections
Radiation source: fine-focus sealed tube	1263 reflections with *I* > 2σ(*I*)
graphite	*R*_int_ = 0.036
phi and ω scans	θ_max_ = 27.6°, θ_min_ = 2.4°
Absorption correction: multi-scan (*SADABS*; Bruker, 2000)	*h* = −19→19
*T*_min_ = 0.011, *T*_max_ = 0.133	*k* = −10→10
10792 measured reflections	*l* = −13→13

### Refinement

**Table:**

Refinement on *F*^2^	Primary atom site location: structure-invariant direct methods
Least-squares matrix: full	Secondary atom site location: difference Fourier map
*R*[*F*^2^ > 2σ(*F*^2^)] = 0.025	Hydrogen site location: inferred from neighbouring sites
*wR*(*F*^2^) = 0.056	H-atom parameters constrained
*S* = 1.11	*w* = 1/[σ^2^(*F*_o_^2^) + (0.016*P*)^2^ + 2.8133*P*] where *P* = (*F*_o_^2^ + 2*F*_c_^2^)/3
1607 reflections	(Δ/σ)_max_ = 0.001
61 parameters	Δρ_max_ = 0.86 e Å^−^^3^
1 restraint	Δρ_min_ = −1.05 e Å^−^^3^

### Special details

**Table:**

Geometry. All e.s.d.'s (except the e.s.d. in the dihedral angle between two l.s. planes) are estimated using the full covariance matrix. The cell e.s.d.'s are taken into account individually in the estimation of e.s.d.'s in distances, angles and torsion angles; correlations between e.s.d.'s in cell parameters are only used when they are defined by crystal symmetry. An approximate (isotropic) treatment of cell e.s.d.'s is used for estimating e.s.d.'s involving l.s. planes.
Refinement. Refinement of *F*^2^ against ALL reflections. The weighted *R*-factor *wR* and goodness of fit *S* are based on *F*^2^, conventional *R*-factors *R* are based on *F*, with *F* set to zero for negative *F*^2^. The threshold expression of *F*^2^ > σ(*F*^2^) is used only for calculating *R*-factors(gt) *etc*. and is not relevant to the choice of reflections for refinement. *R*-factors based on *F*^2^ are statistically about twice as large as those based on *F*, and *R*- factors based on ALL data will be even larger.

### Fractional atomic coordinates and isotropic or equivalent isotropic displacement parameters (Å^2^)

**Table:**

	*x*	*y*	*z*	*U*_iso_*/*U*_eq_	Occ. (<1)
Pb1	0.5000	0.5000	0.5000	0.05028 (11)	
I1	0.66188 (4)	0.2500	0.44218 (7)	0.06995 (19)	
I2	0.45861 (4)	0.2500	0.73162 (5)	0.05961 (16)	
I3	0.38187 (5)	0.2500	0.33215 (6)	0.06837 (19)	
C1	0.0996 (6)	0.1682 (10)	0.4247 (8)	0.100 (3)	
H1	0.0708	0.1100	0.3609	0.120*	
C2	0.1419 (7)	0.0883 (12)	0.5186 (9)	0.103 (3)	
H2	0.1424	−0.0261	0.5197	0.123*	
C3	0.1824 (5)	0.1708 (11)	0.6087 (7)	0.100 (3)	0.50
H3	0.2114	0.1142	0.6731	0.120*	0.50
N1	0.1824 (5)	0.1708 (11)	0.6087 (7)	0.100 (3)	0.50
N2	0.2230 (10)	0.132 (2)	0.7177 (16)	0.121 (5)	0.50
H2A	0.2415	0.2091	0.7672	0.146*	0.50
H2B	0.2307	0.0310	0.7382	0.146*	0.50

### Atomic displacement parameters (Å^2^)

**Table:**

	*U*^11^	*U*^22^	*U*^33^	*U*^12^	*U*^13^	*U*^23^
Pb1	0.0585 (2)	0.03787 (16)	0.05448 (19)	0.00017 (13)	0.00130 (15)	−0.00097 (14)
I1	0.0549 (3)	0.0611 (4)	0.0938 (5)	0.000	0.0189 (3)	0.000
I2	0.0727 (4)	0.0575 (3)	0.0486 (3)	0.000	0.0023 (3)	0.000
I3	0.0753 (4)	0.0603 (4)	0.0696 (4)	0.000	−0.0268 (3)	0.000
C1	0.120 (7)	0.100 (6)	0.081 (5)	−0.001 (5)	−0.015 (5)	−0.010 (5)
C2	0.104 (7)	0.089 (6)	0.114 (8)	−0.005 (5)	0.008 (6)	0.004 (6)
C3	0.064 (4)	0.162 (9)	0.074 (5)	0.010 (4)	0.010 (3)	0.026 (5)
N1	0.064 (4)	0.162 (9)	0.074 (5)	0.010 (4)	0.010 (3)	0.026 (5)
N2	0.111 (11)	0.121 (12)	0.133 (13)	0.004 (10)	−0.004 (11)	0.029 (11)

### Geometric parameters (Å, °)

**Table:**

Pb1—I3^i^	3.2301 (5)	C1—C1^iii^	1.331 (16)
Pb1—I3	3.2301 (5)	C1—C2	1.345 (12)
Pb1—I1	3.2303 (5)	C1—H1	0.9300
Pb1—I1^i^	3.2303 (5)	C2—C3	1.315 (11)
Pb1—I2	3.2412 (5)	C2—H2	0.9300
Pb1—I2^i^	3.2412 (5)	C3—C3^iii^	1.288 (18)
I1—Pb1^ii^	3.2303 (5)	C3—H3	0.9300
I2—Pb1^ii^	3.2412 (5)	N2—H2A	0.8600
I3—Pb1^ii^	3.2301 (5)	N2—H2B	0.8600
			
I3^i^—Pb1—I3	180.0	I2—Pb1—I2^i^	180.0
I3^i^—Pb1—I1	94.886 (17)	Pb1^ii^—I1—Pb1	78.000 (17)
I3—Pb1—I1	85.114 (17)	Pb1^ii^—I2—Pb1	77.688 (16)
I3^i^—Pb1—I1^i^	85.114 (17)	Pb1—I3—Pb1^ii^	78.007 (16)
I3—Pb1—I1^i^	94.886 (17)	C1^iii^—C1—C2	118.9 (6)
I1—Pb1—I1^i^	180.0	C1^iii^—C1—H1	120.6
I3^i^—Pb1—I2	94.940 (16)	C2—C1—H1	120.6
I3—Pb1—I2	85.060 (16)	C3—C2—C1	120.4 (9)
I1—Pb1—I2	83.844 (15)	C3—C2—H2	119.8
I1^i^—Pb1—I2	96.156 (15)	C1—C2—H2	119.8
I3^i^—Pb1—I2^i^	85.060 (16)	C3^iii^—C3—C2	120.7 (6)
I3—Pb1—I2^i^	94.940 (16)	C3^iii^—C3—H3	119.7
I1—Pb1—I2^i^	96.156 (15)	C2—C3—H3	119.7
I1^i^—Pb1—I2^i^	83.844 (15)	H2A—N2—H2B	120.0

Symmetry codes: (i) −*x*+1, −*y*+1, −*z*+1; (ii) −*x*+1, *y*−1/2, −*z*+1; (iii) *x*, −*y*+1/2, *z*.
